# Cardiovascular changes after pneumonia in a dual disease mouse model

**DOI:** 10.1038/s41598-022-15507-w

**Published:** 2022-07-01

**Authors:** Benjamin Bartlett, Herbert P. Ludewick, Shipra Verma, Vicente F. Corrales-Medina, Grant Waterer, Silvia Lee, Girish Dwivedi

**Affiliations:** 1 Department of Advanced Clinical and Translational Cardiovascular Imaging, Harry Perkins Institute of Medical Research, Murdoch, Australia; 2 School of Medicine, University of Western Australia, Perth, WA Australia; 3 Department of Cardiology, Fiona Stanley Hospital, Murdoch, WA Australia; 4 Department of Microbiology, Pathwest Laboratory Medicine, Perth, Australia; 5 Department of Nuclear Medicine, PET CT and Radionuclide Therapy, Fiona Stanley Hospital, Murdoch, WA Australia; 6 Department of Geriatric Medicine, Fiona Stanley Hospital, Murdoch, WA Australia; 7 Department of Medicine, University of Ottawa, Ottawa, Canada; 8 Clinical Epidemiology Program, The Ottawa Hospital Research Institute, Ottawa, Canada; 9 Royal Perth Hospital, Perth, WA Australia; 10 Harry Perkins Institute of Medical Research and Fiona Stanley Hospital, The University of Western Australia, Perth, Australia

**Keywords:** Infection, Animal disease models, Cardiovascular models

## Abstract

Residual inflammation in cardiovascular organs is thought to be one of the catalysts for the increased risk of cardiovascular complications seen following pneumonia. To test this hypothesis, we investigated changes in plaque characteristics and inflammatory features in *ApoE*−/− mouse aorta and heart following pneumonia. Male *ApoE*−/− mice were fed a high fat diet for 8 weeks before intranasal inoculation with either *Streptococcus pneumoniae* serotype 4 (test group) or phosphate buffered saline (control group). Mice were sacrificed at 2-, 7- and 28-days post-challenge. Changes in plaque burden and characteristics in aortic root and thoracic aorta were characterized by Oil red O and Trichrome stains. Inflammatory changes were investigated by FDG-PET imaging and immunofluorescence staining. We found TIGR4-infected mice present with increased plaque presence in the aortic root and thoracic aorta at 2- and 28-days post-inoculation, respectively. Aortic wall remodelling was also more pronounced in mice challenged with pneumococci at 28 days post-inoculation. Aortic root plaques of infected mice had reduced collagen and smooth muscle cells, consistent with an unstable plaque phenotype. Pneumonia alters plaque burden, plaque characteristics, and aortic wall remodelling in *ApoE*−/− mice. These effects caused by *Streptococcus pneumoniae* TIGR4, may contribute to the increased risk of cardiovascular complications seen in survivors of this infection.

## Introduction

Cardiovascular (CV) disease (CVD) is the leading cause of global morbidity and mortality. In Australia, CVD accounts for ~ 27% of all deaths, killing one person every 19 min, or 43,477 people each year^1,2^. There is accumulating evidence of increased short- and long-term risk of adverse CV event following pneumonia^3^. Up to 30% of patients that are hospitalised with community-acquired pneumonia (CAP) develop adverse cardiac events (myocardial infarction, stroke and heart failure) within 30 days after admission^3–5^. After discharge, the risk of developing adverse cardiac events continues to be elevated, and while it is greatest in the first 30 days, it remains increased for up to 10 years post discharge when compared to patients without pneumonia even after adjustment for traditional CV risk factors^6^. The risk of adverse cardiac events increases with the severity of the pneumonia episode^6^. Whilst the mechanisms by which pneumonia can affect long-term risk of CVD are poorly understood, potential mechanistic pathways such as the development of myocarditis^7–9^ and alteration or progression of cellular composition of atherosclerotic plaques making them more vulnerable to complications have been suggested^10–12^. Previously in nonhuman primates and in patient autopsies, *Streptococcus pneumoniae* has been shown capable of translocating to the myocardium to form microlesions and disrupt cardiac function^5,7,13^. Inflammation is now considered central to the development and progression of atherosclerosis^14^ and it has been proposed that persistent inflammatory activity post-pneumonia can mediate any putative effect of pneumonia on atherosclerotic plaques^15^. In pneumonia survivors, lung inflammation persists for several weeks after clinical resolution of the infection, and IL-6 levels at hospital discharge predict CV mortality at 1-year post-discharge^16,17^. While the medium- and long-term CV risk after pneumonia has mostly been assessed in patients without pre-existing clinical CVD^6^, it is intuitive that the risk in patients with pre-established plaques will be higher^18–20^. In this study, we used a previously developed in-house dual disease mouse model of pneumonia and atherosclerosis^21^, and investigated plaque burden and characteristics, and inflammatory changes in the aorta and heart that can potentially contribute to CV complications after this infection.

## Results

### Aortic plaque size and characteristics, and arterial wall remodelling

**Figure 1:**
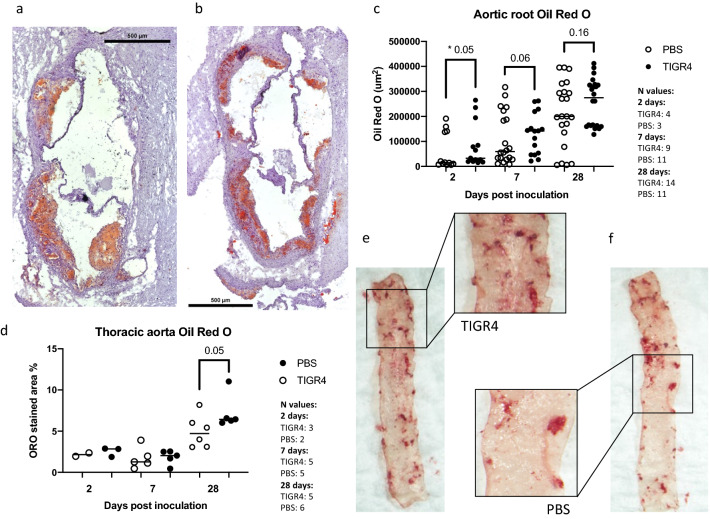
Oil Red O staining of aortic root and thoracic aorta. (**a**) and (**b**) are representative images (taken at 10x) of aortic root from TIGR4- and PBS- inoculated mice at 28-days post-inoculation (PI). (**c**) The area of Oil Red O staining was quantified and compared between groups. Significantly more plaques were observed 2 days post TIGR4 inoculation. (**d**) Quantification of Oil Red O content in thoracic aortas as a percentage of the area of tissue at 2-, 7- and 28- days PI. At 28 days PI, TIGR4 inoculated mice present with significantly more plaque in the thoracic aorta compared to PBS control. (**e**) and (**f**) are representative images of thoracic aorta from TIGR4- and PBS-inoculated mice, respectively.

To determine if infection with TIGR4 had any impact on lipid content and plaque size, aortic root sections were stained with Oil Red O (Fig. 1a,b). On Oil red staining of the aortic root, there was significantly more plaque in the aortic root of TIGR4-inoculated mice compared to phosphate buffered saline (PBS)-inoculated mice at 2 days post-inoculation (PI) (p = 0.05, Fig. 1c), but no significances were observed at 7- and 28-days PI. Assessment of plaque as a percentage of tissue in the thoracic aorta revealed significantly more plaque at 28 days (but no 2 and 7 days) PI in the TIGR4-inoculated mice compared to the PBS-inoculated animals (p = 0.05) (Fig. 1d–f).

**Figure 2:**
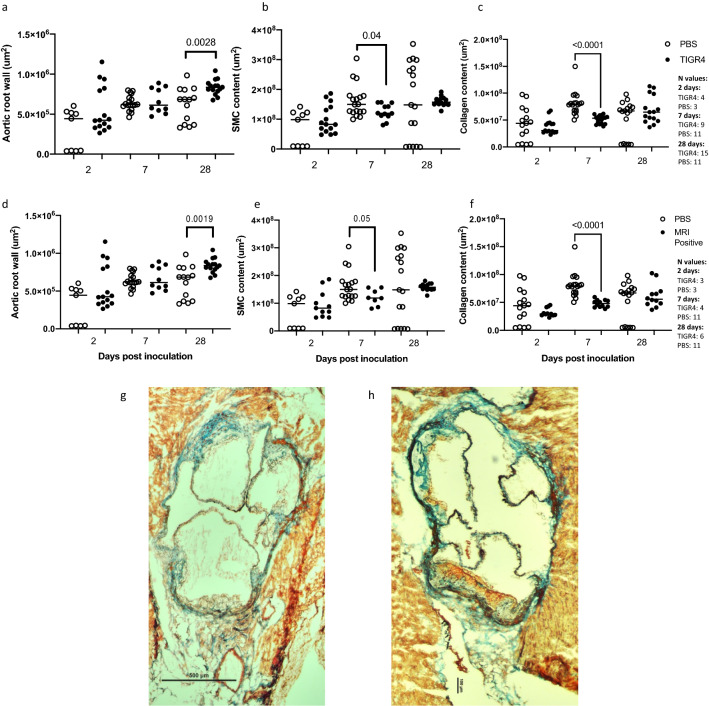
Assessment of aortic root remodelling and plaque structure using trichrome staining. (**a**) Total area of aortic wall was quantified to assess inward and outward remodeling as a result of atherosclerosis. 28 days post-inoculation (PI), TIGR4 inoculated mice present with significantly more aortic wall remodelling. 8 μm tissue sections were stained with modified trichrome stain to identify collagen and smooth muscle cells (SMC). Following colour deconvolution, SMC (**b**) and collagen (**c**) intensity per area in aortic root were calculated. Significantly less SMC and collagen were present in the plaques of TIGR4 inoculated mice compared to PBS inoculated mice at 7 days PI. Moreover, aortic remodelling, plaque-SMC and -collagen content in TIGR4-inoculated mice with confirmed pulmonary abnormalities (MRI positive) were compared to PBS-inoculated mice (**d**), (**e**) and (**f**), respectively. Similar significant comparisons were observed. (**g**) and (**h**) are representative images of aortic root from TIGR4- and PBS-inoculated mice, respectively (taken at 10x).

Tissues were stained with trichrome to investigate if infection with TIGR4 affected the plaque composition of smooth muscle cells (SMC) and collagen in the aortic root (Fig. 2g,h). Quantification of SMC (red) and collagen (green) intensity/area demonstrated significant reduction in SMC and collagen content in plaques at 7 days PI in TIGR4-inoculated mice compared to PBS-inoculated mice (p = 0.04 and p < 0.0001, Fig. 2b,c). No differences were evident at 2- and 28-days PI. In TIGR4-infected mice with evidence of pulmonary infection, there was a significant reduction in SMC (p = 0.05) and collagen (p < 0.0001) content within plaques in the aortic root compared to PBS-inoculated mice at 7 days PI (Fig. 2e,f).

To investigate the effect of TIGR4 infection on artery wall remodelling, aortic root area was quantified in tissues stained with trichrome using ImageJ software. A significant difference was evident at 28 days PI, with TIGR4-inoculated mice having increased aortic root wall area compared to PBS-inoculated mice (p = 0.0028, Fig. 2a). TIGR4-inoculated mice with evidence of pulmonary infection presented with more severe remodelling compared to PBS-inoculated mice at 28 days PI (p = 0.0019, Fig. 2d).

### Inflammatory markers in the aortic root following TIGR4 infection

**Figure 3:**
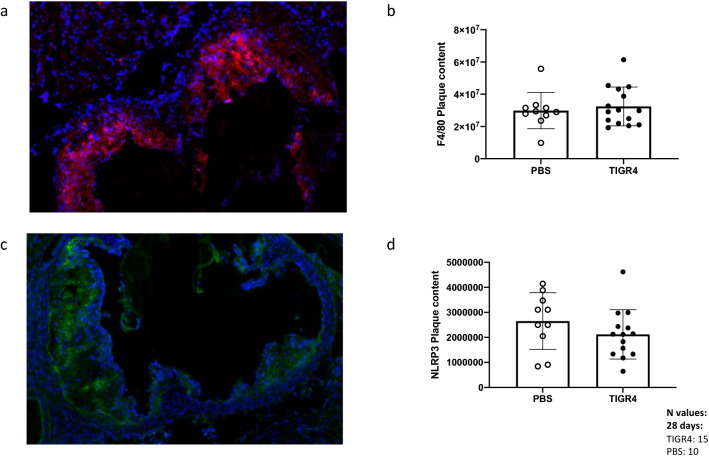
Immunofluorescence staining of aortic root. (**a**) A representative image (taken at 10x) of aortic root stained for F4/80 (red) and DAPI (blue) from TIGR4- and PBS- inoculated mice at 28-days post-inoculation. (**b**) Red fluorescence intensity was quantified per area in plaque regions (minus any background fluorescence detected) and compared between groups. (**c**) Representative image (taken at 10x) of aortic root stained for NLRP3 (green) and DAPI (blue) from TIGR4- and PBS- inoculated mice at 28-days post-inoculation. (**d**) Green fluorescence intensity was quantified per area in plaque regions (minus any background fluorescence detected) and compared between groups. No significance was observed.

As significant differences in remodelling were observed only at 28 days PI, immunofluorescence staining was performed at this time-point to identify macrophages (Fig. 3a) and inflammasome activation (Fig. 3c). Quantitation of F4/80 and NLRP3 were expressed as staining intensity per area. Intraplaque macrophage content (Fig. 3b) and NLRP3 inflammasome staining (Fig. 3d) were comparable in TIGR4- and PBS-inoculated mice. Furthermore, F4/80 and NLRP3 staining did not differ between TIGR4-inoculated mice with pulmonary infection and PBS-inoculated mice (Supplementary Fig. 2).

### FDG uptake in aortic arch

**Figure 4:**
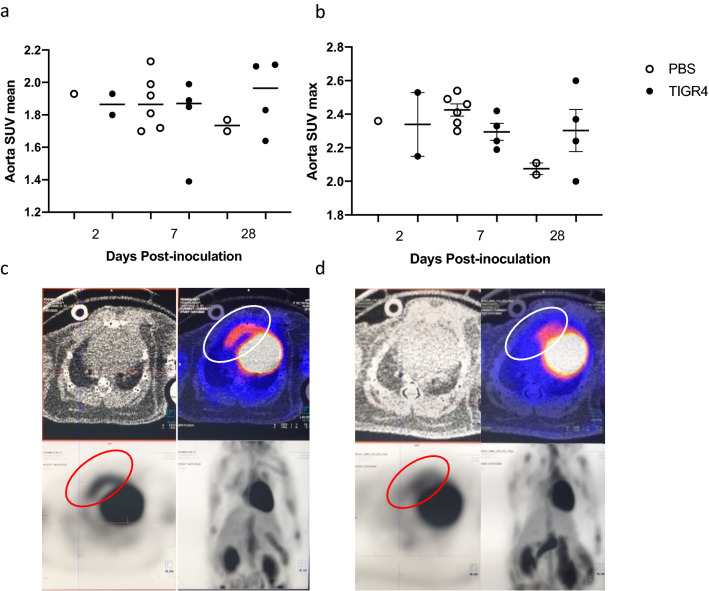
FDG uptake in the aortic arch. TIGR4- and PBS-inoculated mice underwent fluorodeoxyglucose positron emission tomography (FDG-PET) imaging at 2-, 7- or 28-days post-inoculation. The FDG standardised uptake value (SUV)-mean (**a**) and -max (**b**) were evaluated for each mouse. No significance was observed. Representative images of TIGR4- (**c**) and PBS-inoculated mice (**d**), respectively. Sagittal views with the red and white circles present fluorodeoxyglucose uptake in the ascending aorta.

FDG uptake was assessed to determine degree of inflammation in the aortic arch from TIGR4-inoculated (n = 10) and PBS-inoculated (n = 9) mice. Consistent with the findings from the immunofluorescence staining, there were no significant changes in FDG standardised uptake values (SUV) across all time-points (Fig. 4a,b).

### Tenascin C (TNC) mRNA expression and microlesion formation in the heart apex

**Figure 5:**
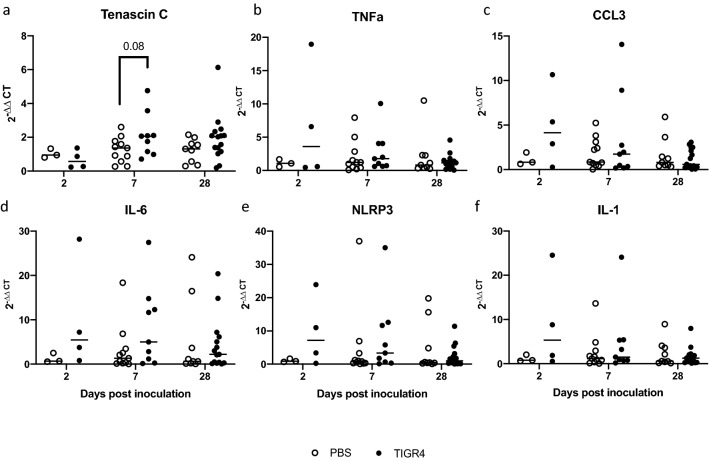
Gene expression of inflammatory mediators in the heart apex. Tissues from TIGR4- and PBS-inoculated mice were collected at 2-, 7- or 28-days post-inoculation and mRNA expression levels of (**a**) tenascin C; at 7 days post-inoculation, there was a trend for increased levels of tenascin C (**b**) tumor necrosis factor-alpha (TNF-a), (**c**) chemokine (C-C motif) ligand 3 (CCL3), (**d**) interleukin-6, (**e**) NLR family pyrin domain containing 3 (NLRP3) and (**f**) interleukin-1 were quantified by real-time polymerase chain reaction. Target gene was normalized against the housekeeping gene, HPRT. Solid horizontal line represents the median.

At 7 days PI, there was a trend for increased mRNA levels of TNC (Fig. 5a, p = 0.08) in the heart apex of TIGR4-inoculated mice compared to PBS-inoculated mice. However, there were no significant differences in the mRNA levels of IL-6, TNFα, CCL3, NLRP3 and IL-1 (Fig. 5b–f). No significant differences were observed in heart apex gene expression when comparing TIGR4-inoculated mice with evidence of pulmonary infection to PBS-inoculated mice (Supplementary Fig. 3). Additionally, there was no evidence of microlesion formation seen in mouse heart via hematoxylin and eosin staining (Supplementary Fig. 4).

## Discussion

To date, the study of the potential mechanisms behind the increase in CV risk after pneumonia in humans has been limited by the lack of validated animal models that would resemble, in one same model, the processes of both atherosclerotic disease and lung infection. *ApoE*−/− mice are a validated model for human atherosclerosis, and bacterial intranasal inoculation is a well validated model for human respiratory infections^7,13,22–25^. We established a dual disease animal model that utilises intranasal inoculation of low dose *S. pneumoniae* in *ApoE*−/− mice to investigate the effect of pneumonia in atherosclerosis progression^21^. In our model, we have previously shown that inoculation of *S. pneumoniae* in *ApoE*−/− mice resulted in a significant proportion (48%) of these animals developing changes consistent with pneumonia on magnetic resonance imaging (MRI), and by limiting our analyses only to those inoculated mice that survive the *S. pneumoniae* challenge, our model more likely resembled the non-fatal forms of pneumonia in humans^21^. Challenging *ApoE*−/− mice with intranasal *S. pneumoniae* in our model resulted in subsequent increases in the atherosclerotic plaque burden of their aortic roots (at 2 days PI) and thoracic aortas (at 28 days PI), and more conspicuous characteristics of instability in their atherosclerotic plaques (reduced plaque SMC and collagen content at 7 days PI) compared to animals challenged with PBS. Hence, this novel twin model of diseases (non-fatal pneumonia and atherosclerosis) proves useful for the study of potential mechanisms by which pneumonia can affect progression of atherosclerosis and increased CV risk after recovering from pneumonia in humans^6^.

We also found evidence of increased arterial wall remodelling (increased aortic root wall area, as measured using ImageJ) at 28 days PI in TIGR4-inoculated mice. Glagov’s phenomenon states that in response to plaque development, arteries undergo remodelling to maintain flow despite increases in atherosclerotic plaque mass^26^. Narrowing of the artery lumen is not simply the result of plaque growth but also failure of the artery wall to maintain appropriate lumen size needed to allow normal blood flow; hence, remodelling may be beneficial, at least in the short-term^27^. It has been suggested that the inability of vessels to remodel is a form of “vascular failure”, similar to the syndrome cardiac failure^27^. However, in the long-term, arterial remodelling involves matrix degradation which may also contribute to the decrease in SMC and collagen content observed in the aortic plaque following intranasal challenge with TIGR4.

While observations in this study did not translate to increased inflammation (macrophages or NLRP3 inflammasome activation) or change in SUV (max and mean) uptake in the aorta, heart apex gene inflammatory gene expression of TNC trended higher in TIGR4-inoculated mice across all time-points compared to PBS-inoculated mice. TNC is a transiently expressed extracellular matrix protein however, under pathological conditions including infection, inflammation, injury, and remodelling, TNC expression can be upregulated^28^. TNC^+/+^ C57Bl/6 mice infected with *Klebsiella pneumoniae* presented with elevated pulmonary TNC protein levels 42 h following infection, and comparably higher local immune response compared to TNC^−/−^ C57Bl/6 mice^29^. In myocardial infarction patients, increased levels of circulating TNC levels have been correlated with high risk of left ventricular remodelling, poor prognosis and mortality^30,31^. Moreover, a significant relationship between the severity of coronary artery disease (by the Gensini score) and TNC levels in patient serum has been observed^32^. Studies have found TNC to be upregulated in plaque with preferential enrichment around the lipid core, shoulder regions and rupture areas^33–35^, thereby driving SMC changes from a non-proliferative phenotype to a migratory synthetic state resulting in plaque development and progression^36^. In addition, TNC has been shown to form a positive feedback loop with matrix metalloproteinases to influence plaque characteristics favouring destabilization and plaque rupture^37^.

Unlike the previous studies, microlesions in the apex of the heart were not observed in TIGR4-inoculated mice^7,8^. Potential explanations of the divergent result include, the longitudinal nature of our respiratory infection model versus the acute model of systemic infection used in previous studies and the fact that microlesion formation seen in previous studies was associated with more severe pneumococcal infection following intraperitoneal injection while our model was established after intranasal infection^7,8^.

While our study sets to investigate the CV changes imposed by *S.pneumoniae* TIGR4, the study was conducted without a wild-type mouse control. The addition of a C57Bl/6 wild-type, while without plaque presence, would enable other inflammatory observations (heart gene expression, systemic inflammation and aortic wall remodelling). Other limitations are more specific to plaque rupture research or the numbers at an earlier time points in the study. As a PBS control mouse presented with a genetic heart defect and a mice succumbed to the infection, the statistical power of the study may have been affected at an earlier time points. However, findings in the remaining mice (especially at 28 days PI) present strong evidence of putative pathways for CV complications following infection. The lack of research that prospectively links factors that cause changes in plaque morphology and rupture progressing to subsequent CV event is an area that has not been studied well. In consideration of this, our study assesses a range of attributes associated with accelerated atherosclerosis and progression to plaque vulnerability, providing comparison between post-infection and PBS-inoculated mice to highlight plaque characteristics and inflammatory changes in the aorta and heart imposed by a low-dose pneumonia infection. In a comparable *ApoE−/−* mouse study, vulnerable plaque was assessed by oxidative stress based on plaque oxygen concentration^38^. The study utilised ratiometric semiconducting polymer nanoparticle (RSPN) photoacoustic imaging to compare lipopolysaccharide caused pneumonia in *ApoE*−/− mice to non-infected *ApoE*−/− mice. The imaging utility was able to reliably identify infected from non-infected based on the oxygen reading. This could be a utility which, in combination with the methods utilised in this paper, may contribute toward developing a more accurate profile of vulnerable plaque.

## Future directions and conclusions

Here, we describe a mouse model to investigate changes in CV characteristics following pneumonia^21^. Future studies can use the same methodology to investigate the role of TIGR4 inoculation in female *ApoE*−/− mice and investigate its impact on CV characteristics, including heart function.

Our study suggests that a delayed accelerating effect on atherosclerosis progression secondary to *S. pneumoniae* infection has the potential to contribute to the increased risk of CV complications that is seen among pneumonia survivors. Our study provides evidence that non-fatal pneumonia infection can alter plaque presence and characteristics that may make plaques more vulnerable to complications seen after CAP. This process also manifests as increased remodelling in the aortic root wall of the infected mice.

## Materials and methods

### Animal model

As previously described, a pneumonia-atherosclerosis dual disease animal model was first established to conduct our experiment^21^. Briefly, two groups of ApoE−/− male mice aged 6–7 weeks were obtained from the Animal Resources Centre (Supplementary Fig. 1a,b) (West Australia, Australia) and maintained under pathogen-free conditions. Male mice were used for the study due to evidence of their higher survival rate following infection^39,40^. Animals were acclimated for 7 days with standard rodent chow before they were transitioned to a high fat diet (HFD) containing 21% fat and 0.15% cholesterol to accelerate development of atherosclerosis (Specialty Feeds, Glen Forest, WA, Australia). After 8 weeks of HFD, *ApoE*−/− mice received either a low infectious dose [10^5^ colony forming units in 40 μl] of *Streptococcus pneumoniae* (TIGR4 strain) or 40 μl of phosphate buffered saline (PBS) (Sigma, Australia) via intranasal inoculation. A group of 30 TIGR4-inoculated and 25 PBS-inoculated mice underwent magnetic resonance imaging (MRI) after 2-, 7-, or 28-days post-inoculation (PI) (Supplementary Fig. 1a). In this model, 13/27 (48%) TIGR4-inoculated mice presented with varying degrees of lung consolidation detected by MRI and significantly higher levels of inflammatory markers (IL-6 and CCL3) in infected mice compared to control mice at 28 days^21^. As further investigation, mice that demonstrated MRI confirmed pneumonia were subsetted and investigated for more severe changes compared to control PBS-inoculated mice. After 2-, 7-, or 28-days PI, thoracic aorta, and aortic root and heart apex tissues were collected. The study was approved by the Animal Ethics Committee at University of Western Australia (F71731) and Harry Perkins Institute for Medical Research (AE114).

### Positron emission tomography (PET)

A group of 10 TIGR4-inoculated and 10 PBS-inoculated mice underwent PET after 2-, 7-, or 28-days PI (Supplementary Fig. 1b). One PBS inoculated mouse was excluded from the study following diagnosis of a genetic heart defect, confirmed by MRI. Mice were fasted (water available) overnight prior to imaging. The mice were warmed for 30 min at ~ 30 °C and isoflurane anaesthesia was administered prior to intravenous (IV) injection of ~ 20 MBq of fluorodeoxyglucose (FDG) in a volume less than 200 μl^41^. Mice were kept warm during the uptake phase of 60 min under anaesthesia before cervical dislocation and part body PET-computed tomography (CT) scan on the BioPET/CT 105 camera (NSW, Australia) with a spatial resolution of 1.02 mm^41^. InVivoScope software (Bioscan Inc.) and Syngio.via software (Siemens Healthineers, Australia) was used for image capture and analysis, respectively. Accounting for decay over time, standardised uptake value (SUV) analysis was calculated as radioactive uptake intensity per region of interest^42^. PET/CT scans of mouse aortas were evaluated by a trained specialist who was blinded to the study groups and designations.

### Histological analyses

Heart was harvested and cut transversely before the aortic root was embedded in Tissue-Tek OCT (ProScitech, Australia) and immediately frozen to prevent tissue damage. Following a method adopted from Centra et al., serial 8 μm sections of the aortic root were collected in a series over 10 slides with 5 sections per slide^43^. To ensure consistency between animals, sections were assessed from the first appearance of 3 valves and subsequent slides were assigned to a staining method across all animals. Trichrome staining was performed according to the manufacturer’s protocol (Abcam, Australia) with slight modification. Aniline blue was replaced with 0.5% fast green in 70% ethanol for collagen staining. This facilitated red, green and blue colour staining, and subsequent colour deconvolution using ImageJ software (NIH, Bethesda, USA)^44^.

For Oil Red O staining, aortic root sections were fixed for 5 min in 10% formalin (Sigma), rinsed in ddH_2_0, incubated for 5 min in 60% isopropanol (Sigma), and stained with working Oil Red O solution (diluted 3:2 in ddH_2_0) (Sigma) for 10 min. Sections were rinsed in ddH_2_0 and incubated in 60% isopropanol for 2 min, hematoxylin (Abcam) for 10 s and mounted with glycerol (Merck, USA). Sections were imaged using a Nikon Eclipse TE2000-U microscope and stained area was quantitated using Image J software (NIH, Bethesda, USA). All sections stained with trichrome or Oil Red O within a select region were quantified and are shown under the respective inoculation and timepoint. This was to present a more comprehensive review of the disease state of each mouse.

To determine the presence of microlesions in the heart apex, hematoxylin and eosin staining was carried out as per manufacturers protocol (Abcam). Sections were mounted in aqueous mounting medium (Fronine, Australia) and imaged using a Nikon Eclipse TE2000-U microscope.

### Immunofluorescence

#### F4/80 (macrophage staining)

Sections of aortic root were fixed for 10 min in acetone (ThermoFisher Scientific, Australia) at − 20 °C. Sections were washed in ddH_2_0 before blocking with 5% goat serum (Sigma) in Tris buffered saline (TBS) for 1 h at room temperature. Slides were washed 3 times (2 min) in TBS before addition of anti-F4/80 antibody (clone BM8, Abcam) diluted 1:100 in blocking buffer and incubated overnight at 4 °C. Slides were washed 3 times (2 min) in 0.05% Tween (Sigma) TBS (TBST) before addition of goat anti-rat AF594 (1:1000, ThermoFisher Scientific) for 1 h in the dark. Sections were washed 3 times (2 min) with 0.05% TBST and mounted in ProLong™ Gold Antifade with DAPI (ThermoFisher Scientific).

#### NOD-, LRR- and pyrin domain-containing protein 3 (NLRP3) inflammasome staining

The NLRP3 inflammasome links arterial lipid deposition and lipoproteins to the inflammatory responses that drive plaque progression^45^. To quantify NLRP3 plaque presence, sections of aortic root were fixed in 50% methanol (ThermoFisher Scientific) at room temperature before 3 times (5 min) wash with PBS (Sigma). Sections were blocked with 3% bovine serum albumin (BSA) (ThermoFisher Scientific) in 0.1% Triton X-100 (Merck)/PBS at room temperature for 30 min. Sections were washed 3 times (2 min) in PBS before addition of anti-NLRP3 (clone Cryo-2, Adipogen, USA) diluted 1:200 in blocking buffer and incubated overnight at 4 °C. Slides were washed 3 times (5 min) with 0.1% Triton X-100/PBS before addition of donkey anti-rabbit AF488 (1:1000, ThermoFisher Scientific). Slides were washed 3 times (5 min) in 0.1% Triton X-100/PBS before mounting in ProLong™ Gold Antifade with DAPI (ThermoFisher Scientific).

For F4/80 and NLRP3, sections were imaged using a Nikon Eclipse TE2000-U microscope and quantitated using ImageJ software (NIH, Bethesda, USA). F4/80 and NLRP3 were expressed as staining intensity per area.

### Oil Red O staining of thoracic aorta

The complete thoracic and abdominal aorta was harvested from mice and stored in PBS at 4 °C. Prior to staining, excess adipose fat was removed. Aortas were rinsed in ddH_2_0 before soaking in 60% isopropanol (Sigma) for 10 min. Tissues were stained with Oil Red O (Sigma) for 30 min and further washed in 60% isopropanol for 2 min. Aortas were imaged using a Nikon Eclipse TE2000-U microscope. Oil Red O content was quantitated using ImageJ software (NIH, Bethesda, USA) and expressed as a percentage of the total tissue area.

### Quantification of gene expression by real-time Polymerase Chain Reaction (PCR)

To investigate the effect of infection on genes involved in heart remodelling and inflammation, ribonucleic acid (RNA) from heart apex tissues were extracted using Trizol reagent (ThermoFisher Scientific) and reverse transcribed to complementary deoxyribonucleic acid (cDNA) using the Tetro cDNA Synthesis Kit (Meridian Bioscience, USA) according to the manufacturer’s instructions. Messenger RNA (mRNA) levels of *hprt* (Mm00446968_m1), *il6* (Mm00446190_m1), *il1* (Mm00434228_m1), *nlrp3* (Mm00840904_m1), *tnfa* (Mm00443258 _m1), Tenascin C (Mm00495662_m1), CCL3 (Mm00441258_m1), were determined by quantitative PCR using Taqman primers and probe (Applied Biosystems, USA) and Taqman Gene Expression Master Mix (Applied Biosystems) on a Rotorgene 6000 (Qiagen, Australia). Cycling conditions for TaqMan PCR were 2 min at 50 °C and 10 min at 95 °C followed by 45 cycles of 15 s at 95 °C and 1 min at 60 °C. Quantitative PCR was performed in triplicate. Data was analysed based on relative expression method with the formula relative expression 2^−ΔΔCT^, where the amount of the target gene was normalized first to the endogenous reference (HPRT) and then relative to a calibrator (control animal).

### Statistics

Data are expressed as median (range). All statistical tests were performed with GraphPad Prism 7 (La Jolla, CA, USA). Mann–Whitney was used to compare two groups. Differences with *p*-values < 0.05 were considered statistically significant.

### Ethics

All animal procedures were carried out in accordance with both the Western Australian Animal Welfare Act and National Institute of Health guidelines. This project and its procedures were approved by the animal ethics committee of the Harry Perkins Institute for Medical Research (AE114) and the University of Western Australia (F71731). The study was carried out in compliance with the ARRIVE guidelines.

## Supplementary Information

Supplementary Figures.

## Data Availability

The datasets used and/or analysed during the current study are available from the corresponding author on reasonable request.
