# Supplementary material for: Cardiovascular changes after pneumonia in a dual disease mouse model
Source: Sci Rep. 2022 Jul 1;12:11124. doi: 10.1038/s41598-022-15507-w (PMC9249762; doi:10.1038/s41598-022-15507-w)
Supplement: Supplementary file 1 — Supplementary Figures. [file 41598_2022_15507_MOESM1_ESM.docx]

**Cardiovascular changes after pneumonia in a dual disease mouse model**

Benjamin Bartlett^1,2^, Herbert P Ludewick^1^, Shipra Verma^5,6^, Vicente F. Corrales-Medina^7,8^, Grant Waterer^2,9^, Silvia Lee^1,4,+^ and Girish Dwivedi^1,2,3,+*^

^1^Department of Advanced Clinical and Translational Cardiovascular Imaging, Harry Perkins Institute of Medical Research, Murdoch, Australia

^2^School of Medicine, University of Western Australia, Australia

^3^Department of Cardiology, Fiona Stanley Hospital, Murdoch, Western Australia, Australia

^4^Department of Microbiology, Pathwest Laboratory Medicine, Perth

^5^Department of Nuclear Medicine, PET CT and Radionuclide Therapy, Fiona Stanley Hospital, Murdoch, Western Australia

^6^Department of Geriatric Medicine, Fiona Stanley Hospital, Murdoch, Western Australia

^7^Department of Medicine, University of Ottawa, Canada,

^8^Clinical Epidemiology Program, The Ottawa Hospital Research Institute, Ottawa, Canada

^9^Royal Perth Hospital, Perth, Western Australia, Australia

^+^Silvia Lee and Girish Dwivedi share senior author and are equal in contribution to this paper.


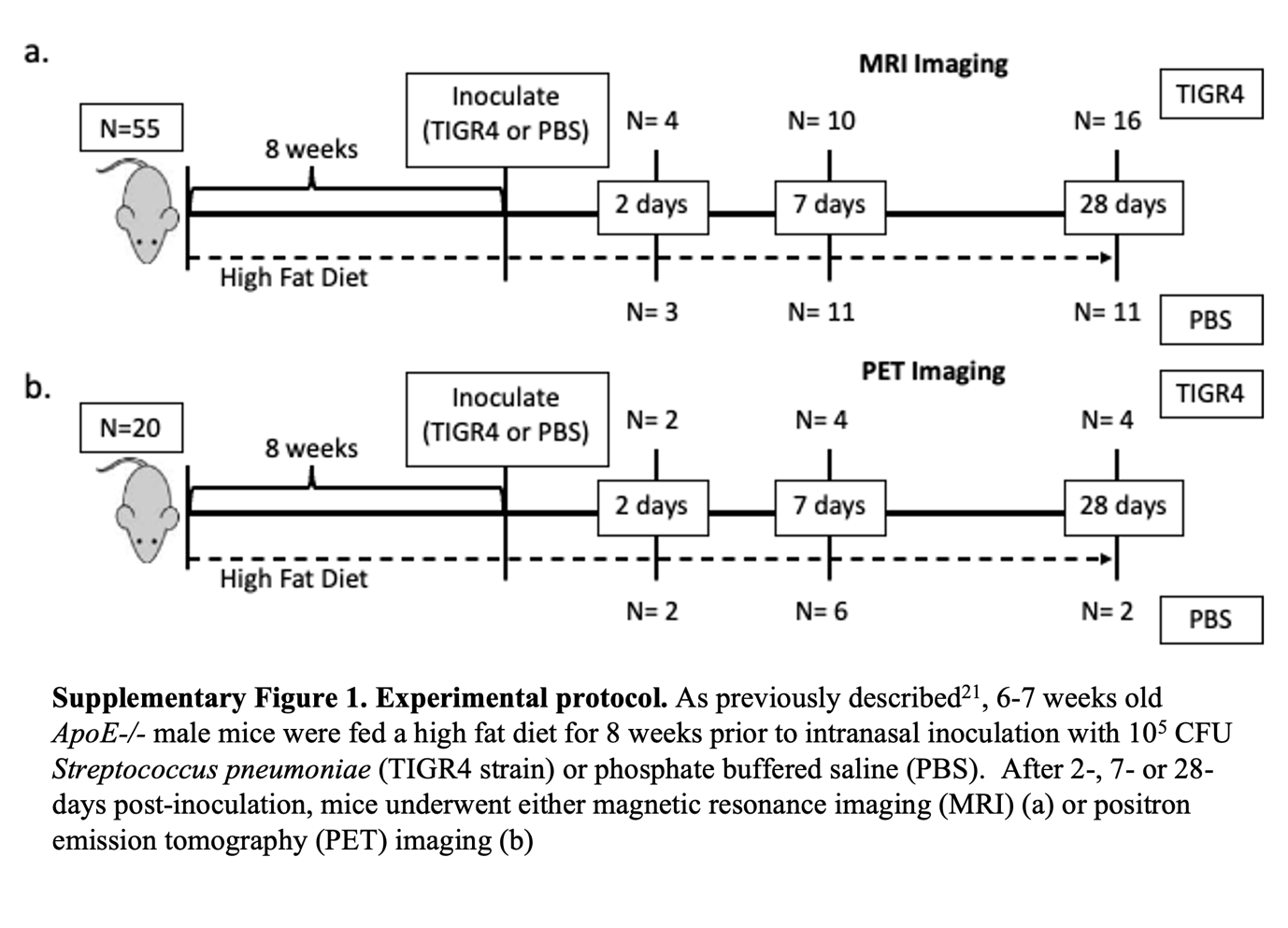


**Supplementary Figure 1. Experimental protocol.** As previously described^21^, 6-7 weeks old

*ApoE-/-* male mice were fed a high fat diet for 8 weeks prior to intranasal inoculation with 10^5^ CFU *Streptococcus pneumoniae* (TIGR4 strain) or phosphate buffered saline (PBS). After 2-, 7- or 28-days post-inoculation, mice underwent either magnetic resonance imaging (MRI) (a) or positron emission tomography (PET) imaging (b).


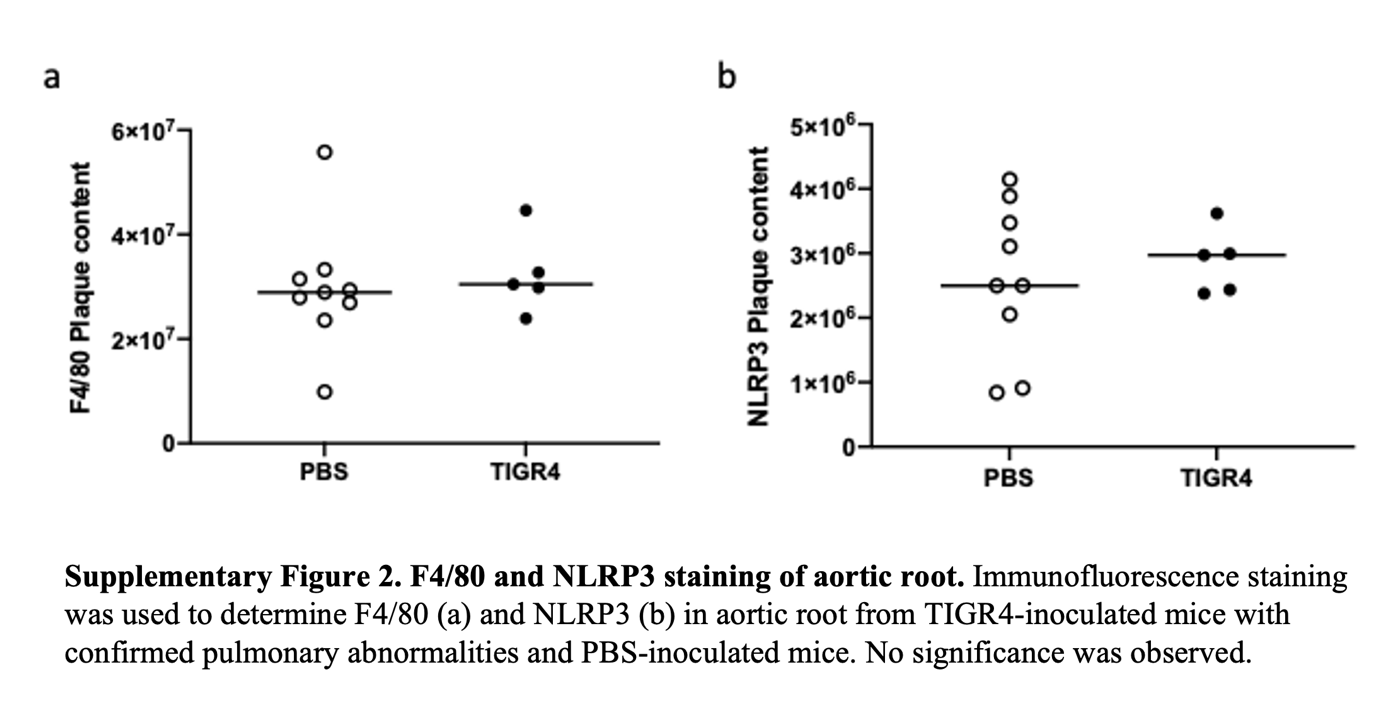


**Supplementary Figure 2. F4/80 and NLRP3 staining of aortic root.** Immunofluorescence staining was used to determine F4/80 (a) and NLRP3 (b) in aortic root from TIGR4-inoculated mice with confirmed pulmonary abnormalities and PBS-inoculated mice. No significance was observed.


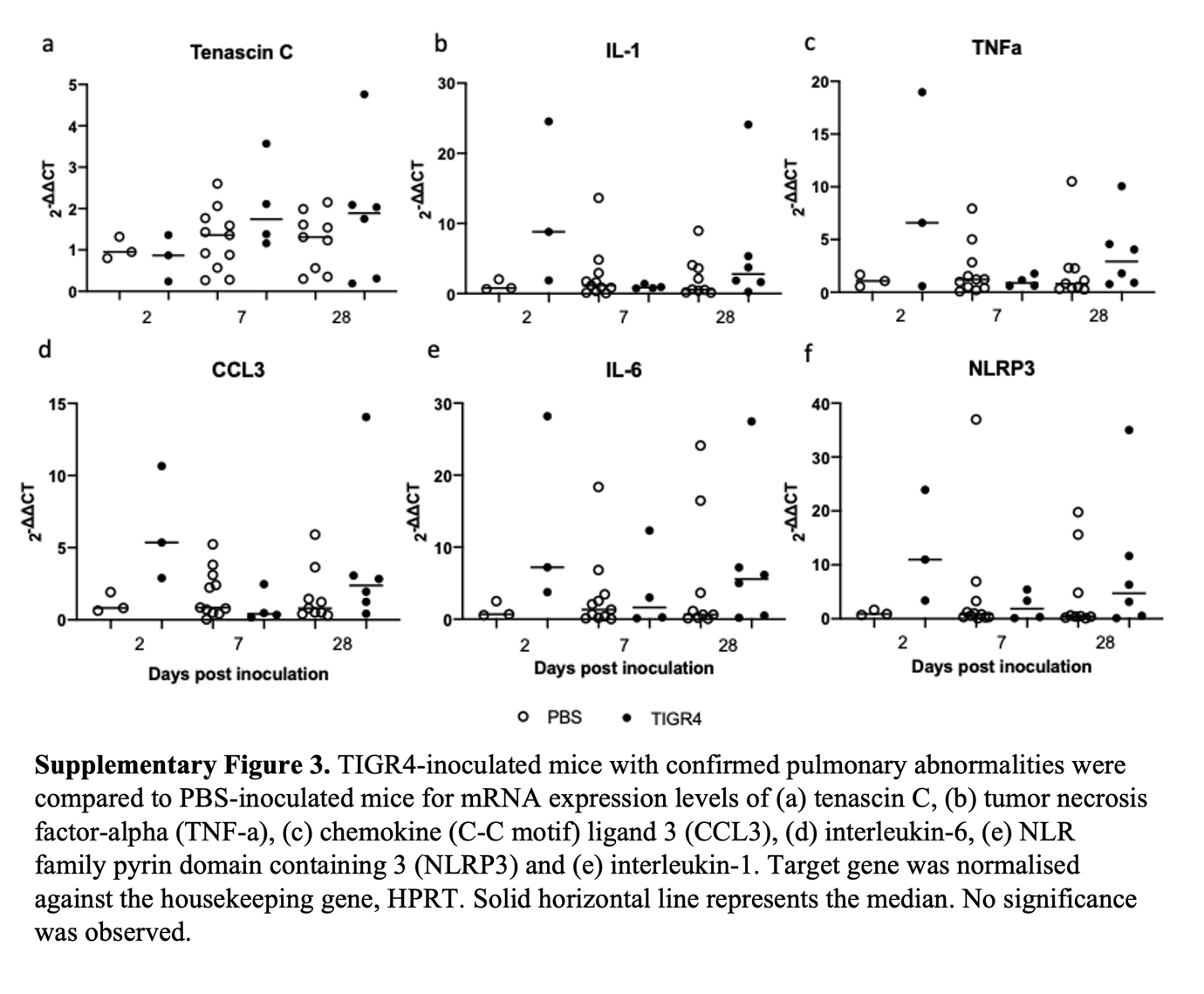


**Supplementary Figure 3.** TIGR4-inoculated mice with confirmed pulmonary abnormalities were compared to PBS-inoculated mice for mRNA expression levels of (a) tenascin C, (b) tumor necrosis factor-alpha )TNF-α), (c) chemokine (C-C motif) legand 3 (CCL3), (d) interleukin-6, (e) NLR family pyrin domain containing 3 (NLRP3) and (e) interleukin-1. Target gene was normalised against the housekeeping gene, HPRT. Solid horizontal line represents the median. No significance was observed.


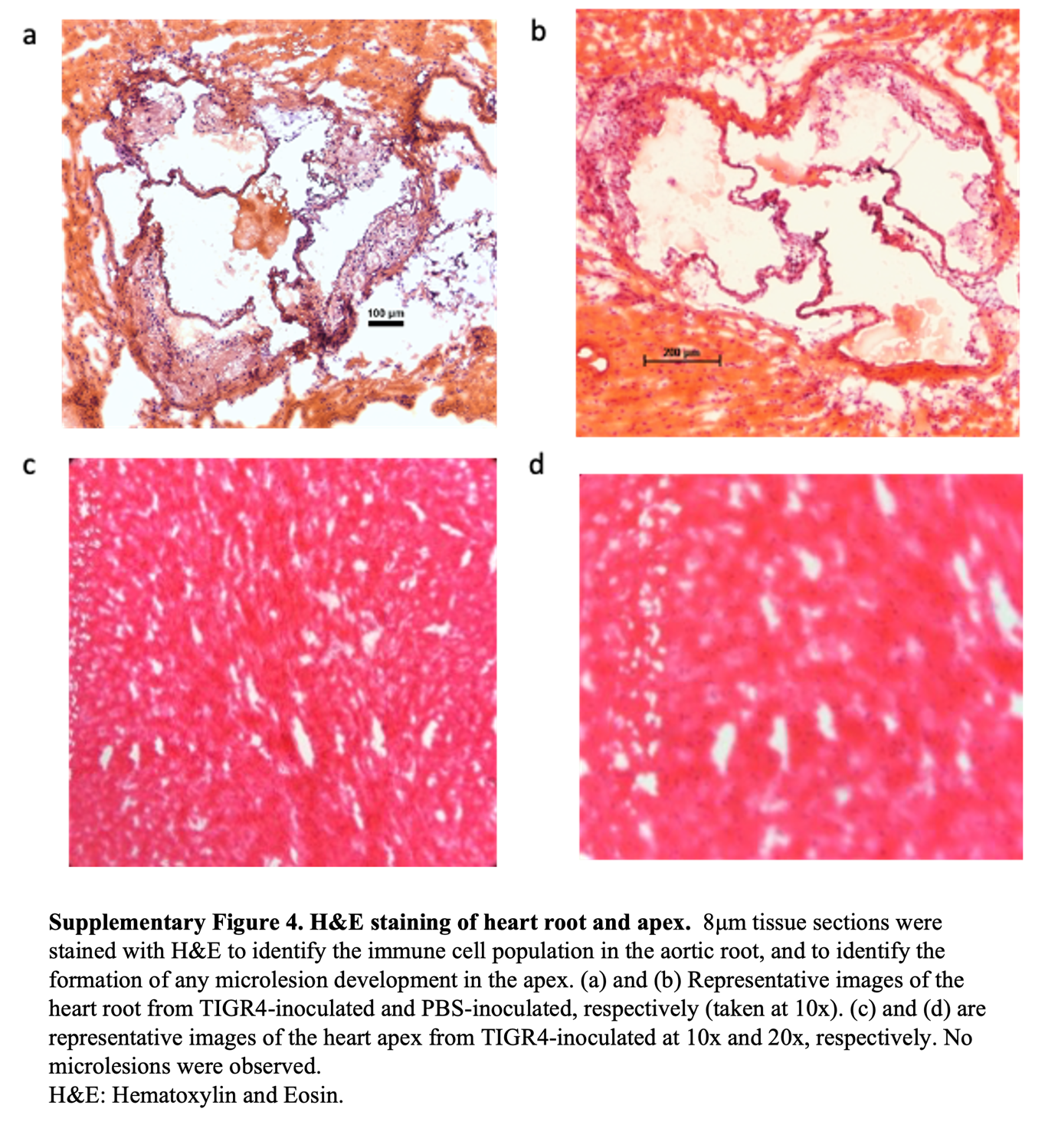


**Supplementary Figure 4. H&E staining of the heart root and apex.** 8μm tissue sections were stained with H&E to identify the immune cell population in the aortic root, and to identify the formation of any microlesion development in the apex. (a) and (b) are representative images of the heart root from TIGR4-inoculated and PBS-inoculated, respectively (taken at 10x). (c) and (d) are representative images of the heart apex from TIGR4-inoculated at 10x and 20x, respectively. No microlesions were observed.

H&E: Hematoxylin and Eosin.
